# Association of Alleles of Human Leukocyte Antigen Class II Genes and Severity of COVID-19 in Patients of the ‘Red Zone’ of the Endocrinology Research Center, Moscow, Russia

**DOI:** 10.3390/diseases10040099

**Published:** 2022-11-02

**Authors:** Ekaterina Troshina, Marina Yukina, Nurana Nuralieva, Evgeny Vasilyev, Olga Rebrova, Ravida Akhmatova, Anna Ikonnikova, Elena Savvateeva, Dmitry Gryadunov, Galina Melnichenko, Natalia Mokrysheva

**Affiliations:** 1 Endocrinology Research Centre, Ministry of Health of the Russian Federation, 117036 Moscow, Russia; 2 Center for Precision Genome Editing and Genetic Technologies for Biomedicine, Engelhardt Institute of Molecular Biology, Russian Academy of Sciences, 119991 Moscow, Russia

**Keywords:** COVID-19, comorbidity, severity of disease, clinical features, HLA class II genes

## Abstract

The aim of this study was to assess the correlations of clinical features of patients with moderate and severe courses of COVID-19, comorbidity (endocrine, autoimmune, cardiovascular, oncological, and pulmonary diseases), and alleles of the HLA class II system genes. One hundred COVID-19 patients hospitalized in the Endocrinology Research Centre, Moscow, Russia, were analyzed for age, gender, smoking, comorbidity, and invasive mechanical ventilation. Computer tomography was used to assess the severity of the disease. HLA-DRB1, HLA-DQA1, and HLA-DQB1 alleles were identified in samples from 100 patients and samples from 327 randomly selected individuals collected in the prepandemic period (control group). There was no association of gender, age, weight, body mass index, smoking, and comorbidity with the severity of COVID-19. Allele DQB1*06:02-8 was more common in patients (*p* < 0.00005), and DQB1*06:01 and DQB1*05:03 were more common in the control group (*p* < 0.00005, and *p* = 0.0011, respectively). DQB1*06:02-8 can probably be considered as predisposing to moderate and severe COVID-19, and DQB1*06:01 can be considered as protective. No association of these alleles with comorbidity was found. Our results suggest that carriers of predisposing alleles, with cardiovascular and non-autoimmune endocrine diseases, should take more stringent preventive measures, and if infected, a more aggressive COVID-19 treatment strategy should be used.

## 1. Introduction

Certain alleles of genes of the human leukocyte antigen (HLA) system are known to increase the risk of pathological activation of the immune system with the development of autoimmune disorders. Alleles of the HLA system genes have also been found to be associated with the risk of developing cardiovascular, pulmonary [1], and cancer diseases, as well as non-autoimmune endocrinopathies [2]. Furthermore, genomics studies have demonstrated strong associations between polymorphisms in major histocompatibility complex (MHC) and HLA genes with susceptibility to infectious diseases [3]. Different HLA genotypes, depending on the degree of induction of the T-cell response, are believed to affect the course of viral infection [4].

Recent data suggest that appropriate innate and adaptive T cell-mediated humoral and cellular immune responses could help the elimination of SARS-CoV-2, which in most cases coincides with clinical recovery [5,6]. On the other hand, an excessive cell-mediated and dysregulated innate and adaptive immune response can lead to an aggressive inflammatory reaction with the release of large amounts of pro-inflammatory cytokines. This condition, known as ‘cytokine storm’—resulting from the excessive production of cytokines by immune cells such as the innate dendritic cells, macrophages, natural killer cells, and the adaptive T and B cells—directly correlates with lung injury, adult respiratory distress syndrome, and multiple organ failure, and leads to an unfavorable prognosis [7,8]. The basic predictive risk factors for cytokine storms in COVID-19 patients include male gender, lactate dehydrogenase level, age over 40 years, the positive test result for replicative SARS-CoV-2 RNA, absolute lymphocyte count, D-dimer and ferritin levels, dynamics in the National Early Warning Score (NEWS) score, and plasma Interleukin 6IL-6 concentration [9]. However, immunogenetic variation in humans could also be an important target for clinical diagnosis and therapeutic intervention of COVID-19 [10].

Determining the effect of HLA gene variations on the course of COVID-19 may help to identify individuals at a higher risk, as well as to understand the different immune responses to the viral infection in the population. Current data about the association between particular HLA alleles/haplotypes and susceptibility or protection against COVID-19 are rather contradictory [4,11,12]. Numerous studies have reported alleles of protection or susceptibility to COVID-19 [13]. In silico analysis of the binding affinity across HLA phenotypes and viral peptides showed that harbors of HLA-B*46:01, HLA-A*11:01, -B*51:01, -C*14:02, HLA-DRB1*15:01, -DQB1*06:02, and -B*27:07 alleles are more vulnerable to SARS-CoV-2, and these mutations predispose patients to a worse disease course [14]. Shkurnikov et al. developed a risk score (RS) which was associated with the ability to present SARS-CoV-2 peptides by the HLA class I molecule set of an individual. An assessment of RS in 111 deceased patients with COVID-19 (Moscow, Russia) and 428 volunteers showed, that the presence of HLA-A*01:01 allele was associated with high risk, while HLA-A*02:01 and HLA-A*03:01 mainly contributed to low risk [15]. Conversely, the study of HLA Class I alleles in the Iranian population did not reveal any association of alleles with COVID-19 severity [16].

Given the more frequent development of severe forms of COVID-19 in patients with an aggravated history (including endocrine, cardiovascular, autoimmune [17], cancer [18], and pulmonary diseases), there may be common predisposing HLA alleles in these conditions. On the other hand, the absence of severe forms of pneumonia in some patients with comorbidities suggests that they have protective alleles. Thus, in a cohort of 306 patients with cancer, mild/moderate COVID-19 was observed in most cases (71%) [19]. It should be taken into account that other factors, such as old age, gender, and obesity, are also associated with an increased severity of the disease [20,21,22,23].

The aim of this study was to assess the correlations of clinical features of patients with moderate and severe courses of COVID-19, comorbid pathology (endocrine, autoimmune, cardiovascular, oncological, and pulmonary diseases), and alleles of the HLA class II system genes.

## 2. Materials and Methods

### 2.1. Patients and Clinical Data

A retrospective study of COVID-19 patients who were hospitalized at the Endocrinology Research Centre in 2020 (detailed patients’ characteristics and procedures are available in the Supplementary Table S1) was conducted. All patients included in the study permanently resided in Moscow or Moscow Region. No other inclusion and exclusion criteria were applied; consecutive sampling was used. The only exclusion criterion was the refusal to participate in the study. The final analysis includes 100 patients.

The diagnosis of COVID-19 was based according to Russian recommendations [24] on radiological signs [25] according to computer tomography (CT) carried out on a Revolution CT (General Electric, Boston, MA, USA) at admission. Repeated CTs were carried out in the case of disease progression and before discharge (1 to 6 times per patient: 1 time in 20% (*n* = 20) of patients; 2 times in 51% (*n* = 51) of patients; 3 times in 22% (*n* = 22) of patients; 4 times in 6% (*n* = 6) of patients; 6 times in 1% (*n* = 1) of patients).

The maximum lung lesion volume during the hospitalization was assessed by CT and qualified (by an expert) as CT-1 for a lesion volume <25% of the lung volume, CT-2 for 25–50% lesion, CT-3 for 51–75% lesion, and CT-4 for >75% lesion [25]. CT-1 and CT-2 cases were considered as a moderate disease, and CT-3 and CT-4 as a severe disease.

In addition to CT signs, all patients had other signs of coronavirus infection before admission, such as a positive test result for SARS-CoV-2 viral RNA in swabs from the nasopharynx and oropharynx confirmed by isothermal amplification (EMG LLC, Innopolis City, Russia) and/or an increase in C-reactive protein (CRP) above 5 mg/L and/or SpO2 below 95% using an Armed YX300 pulse oximeter. The molecular assay used in this study to detect SARS-CoV-2 RNA was validated by comparison with conventional reference real-time PCR (SARS-CoV-2/SARS-CoV Multiplex, DNA-Technology, Russia), with a sensitivity and specificity of 99.0%. The diagnosis of COVID-19 was based on CT data and at least one of the above confirmatory tests.

Two clinical outcomes of COVID-19 were recorded: fatal (death) or improvement. Improvement was defined as the appearance of at least one of the following signs: normalization of CRP (5 mg/L or less), reduction in CT lung lesions, and increase in SpO2 (95% or more). After improvement, the patients were discharged from the hospital; the treatment was continued on an outpatient basis.

The following patient characteristics were analyzed: age, gender, smoking (a patient was considered a smoker if he smoked at least 100 cigarettes during his life and continues to smoke currently [26]), weight, body mass index (BMI), comorbidity according to the patient, and medical documents—endocrine (ED), cardiovascular (CVD), autoimmune (AID), pulmonary (PD), and oncological (OD) diseases; invasive mechanical ventilation (IMV) during hospitalization.

### 2.2. HLA Typing

HLA-DRB1, HLA-DQA1, and HLA-DQB1 alleles were identified in 100 patients and 327 randomly selected individuals in Moscow and Moscow Region (population control group, samples were collected in the prepandemic period and used only for population studies of alleles of HLA class II genes). Genomic DNA was isolated from venous blood lymphocytes. Low-resolution typing of HLA class II (DRB1, DQA1, DQB1) genes was performed using a DT-96 Real-Time PCR thermocycler (DNA-Technology, Russia) and the HLA-DRB1, HLA-DQA1, HLA-DQB1 Real-Time PCR genotyping kits (DNA-Technology, Russia) according to the manufacturer’s instructions. These tests have passed all trials and were approved for in vitro diagnostic by the Federal service for surveillance in the healthcare of the Russian Federation. The diagnostic specificity of the assays, declared by the manufacturer, is 98.7–100% in comparison with the reference sequencing. Genotyping for all loci was performed at the level of allele groups, not specific alleles.

### 2.3. Statistical Analysis

The Statistica v.13 software (TIBCO Software, USA) was used for data analysis. The descriptive statistics are medians (Me), minimum and maximum values for quantitative data, and absolute and relative frequencies for categorical data. The comparison of independent groups was performed by the Mann–Whitney (U) and Kruskal–Wallis (H) tests for quantitative data, Pearson’s Chi-Square, Yates’ adjusted Chi-Square (χ^2^), and two-sided Fisher’s exact test (FET) for categorical data. The odds ratio (OR) and 95% CI were calculated using statistical tools [27,28]. The basic significance level was 0.05, and the Bonferroni correction was applied if necessary. *p*-values between the Bonferroni-corrected level and 0.05 were considered as indicators of statistical tendency.

## 3. Results

Among the patients’ contingent, there were 12% of cases of CT-4, 47% of CT-3, 34% of CT-2, and 7% of CT-1. There were no significant differences in age and gender in the CT groups. The age of females was higher than that of males (*p* = 0.00084, U). There was no significant age difference between severe and moderate COVID-19 (Table 1). A total of 45% of patients were diagnosed with obesity; however, there was no significant difference in BMI and body weight in severe and moderate disease cases. There were also no significant differences between the groups in the frequencies of AID, OD, CVD, PD, and non-autoimmune ED.

IMV was performed in 3% of patients (*n* = 3): two females 75 and 83 years old with CT-4, and one male 67 years old with CT-3. All cases of the disease that required this type of treatment ended in death. There were no lethal outcomes during hospitalization among patients who did not undergo IMV.

The results of HLA-typing (absolute and relative allele frequencies in the entire cohort and in groups) are presented in Table 2. There were no significant differences in HLA class II gene alleles in patients with moderate–severe and severe courses of the Coronavirus infection. The following alleles of the HLA class II genes had frequencies greater than 20%: DQA1*05:01 (30%), DQB1*03:01 (21.5%), and DQB1*06:02-8 (21%) (the corresponding rows are highlighted in bold in Table 2).

Then, a comparative analysis of the frequency of alleles in the examined cohort and in a population control group of individuals from Moscow and the Moscow Region was carried out (Table 3). In the cohort of patients, the frequencies of three alleles of the *DQB1* gene differ from those in the control group: the DQB1*06:02-8 allele was more frequent (RR 5.166, 95% CI (4.525; 5.166)), and the DQB1*06:01 and DQB1*05:03 alleles were less frequent. There were no significant differences in the frequencies of other alleles studied.

The results of the analysis of clinical and genetic characteristics of patients carrying the DQB1*05:03, DQB1*06:01, and DQB1*06:02-8 alleles are shown in Table 4. Patients with a suspected predisposing allele in more than 50% of the cases experienced severe COVID-19, CVD, and non-endocrine AID. One patient with both DQB1*06:01 and DQB1*06:02-8 alleles and moderate COVID-19 (CT-2) at the age of 48 had no comorbidities. The DRB1*15-DQA1*01:02-DQB1*06:02-8 was the most common haplotype in patients with the DQB1*06:02-8 allele.

The association of the COVID-19 severity with allele combinations was checked. To test this hypothesis, the absolute and relative frequencies of HLA haplotypes in the groups were compared. In the general cohort of patients, the predominance of any haplotype was not found (Table 5). The following HLA haplotypes had frequencies 10% or more: DRB1*01-DQA1*01:01-DQB1*05:01 (14%; serotype DR1), DRB1*15-DQA1*01:02-DQB1*06:02-8 (13.5%; serotype DR2), DRB1*11-DQA1*05:01-DQB1*03:01 (12.5%; serotype DR5), and DRB1*03(DRB1*17)- DQA1*05:01-DQB1*02 (10%; serotype DR17).

## 4. Discussion

The median age of hospitalized patients with moderate to severe COVID-19 was 58.5 years. Unlike other authors [29,30,31], we found no age correlation with the severe course of the infection. A slight predominance of males with COVID-19 [32,33,34], most likely associated with smoking, to which they are traditionally more predisposed [35], was also noted by other authors. No significant effect of gender on the severity of COVID-19 was found unlike the result of another Russian study, which included more patients [9].

As in other studies [15,36], the majority of patients in our study had comorbidities, the most common of which in the general cohort were CVD (64% of cases), non-autoimmune ED (59%), and obesity (45%). However, there was no statistically significant effect of the presence of CVD and non-autoimmune ED on the severity of the course of coronavirus infection. Patients with a severe course of the disease were characterized by higher body weight and BMI, which was also emphasized by other authors [37]. However, this correlation was not significant. It is noteworthy that all patients who underwent IMV (all of these cases ended in death) were obese (BMI 32–37.5 kg/m2). However, there was no significant effect of BMI on the severity of the course of coronavirus infection. In contrast to the study by Richardson et al. [38] which indicated a high incidence of type 2 diabetes mellitus in hospitalized patients with COVID-19 (33.8%), we detected this disease in only 12% of the patients.

Less frequently (26%), patients with COVID-19 reported a burdened history of PD without the prevalence of any particular nosology. It is noteworthy that the incidence of tuberculosis in our study was higher (4%) than the incidence of this disease (1%) in the larger cohort (12,513 patients with coronavirus infection) examined by Sy et al. [39]. This may be due to the smaller size of our sample. For this reason, no correlation was found between the presence of PD and the severity of coronavirus infection, unlike other studies [40,41].

OD, compared with CVD, non-autoimmune ED, and PD, occurred less frequently–in 5% of cases. Comparable data were reported by other authors: 3.9% in the study by Singh et al. [36] and 6% in the study by Richardson et al. [38]. The prevalence of the frequency of any OD in the general cohort, as well as its impact on the severity of COVID-19, was not found.

Concomitant AID was detected in 16% of patients. The results of our study did not confirm the earlier assumption about the development of more severe forms of coronavirus infection in patients with AID (OR 0.874, *p* > 0.05), which is consistent with the data of Murtas et al. [42]. These authors reported a low frequency of AID in COVID-19 (3.2%), as well as in other respiratory diseases (3.9%).

For the first time in Russia, the association of the frequency of HLA class II gene polymorphisms with the severity of COVID-19 was investigated (another Russian study [15] evaluated the frequency of HLA class I gene polymorphisms). When comparing the frequency of occurrence of HLA class II alleles in patients with moderate and severe coronavirus infection, no significant differences were found. In the general cohort, the most common allele of the *DQA1* gene was DQA1*05:01, while those of the *DQB1* gene were DQB1*02, DQB1*03:01, DQB1*05:01, and DQB1*06:02-8. As for the *DRB1* gene, no predominance of the frequency of any allele was found, but a high frequency of the DRB1*15 and DRB1*01 alleles was noted. When comparing the frequency of occurrence of alleles in the examined cohort and a population control group, a significant increase in frequency was confirmed only for the DQB1*06:02-8 allele (*p* < 0.00005). This allele has been shown to be associated with narcolepsy and multiple sclerosis [43], but these pathologies were not found in the examined cohort.

In contrast, the incidence of the DQB1*06:01 (*p* < 0.00005) and DQB1*05:03 (*p* = 0.0011) alleles was lower in the group of patients with COVID-19 compared to the population control group. It is noteworthy that these alleles have been shown to be associated with a predisposition to hepatitis B [44]. At the same time, the DQB1*06:02-8 allele is protective against HIV infection (providing a more effective presentation of the CD4+ virus epitope to T-lymphocytes [45]). It is likely that the process of presentation of SARS-CoV-2 antigens, due to the features of the molecular structure of the virus, differs from that of hepatitis B and HIV [4].

Thus, DQB1*06:02-8 can probably be considered as predisposing to moderate and severe COVID-19, and DQB1*06:01 can be considered as protective. Moreover, the DQB1*06:02-8 allele was one of the most frequent among other alleles of the *DQB1* gene in the examined cohort of patients; it was also significantly more common in the examined cohort than in the population control group of Moscow and the Moscow Region. Since the DQB1*05:03 allele was detected only in one patient with severe COVID-19, it was impossible to draw any definite conclusion about its protective effect. Currently, we can only state that this allele has a decreased frequency of occurrence in severe COVID-19.

Our results suggest that carriers of alleles that may be associated with a high risk of developing moderate and severe forms of the disease, who have comorbidities such as CVD, non-autoimmune ED, and, especially, obesity, should take more stringent preventive measures, be considered as priority candidates for vaccination, and if infected, a more aggressive COVID-19 treatment strategy should be used. In order to confirm the presented data, as well as to continue the study of the clinical and genetic characteristics of coronavirus infection, further research on patients with mild and asymptomatic COVID-19 is required. When comparing the frequency of occurrence of HLA haplotypes in the groups, no significant differences were found. In the general cohort of patients, the following HLA haplotypes were more common: DRB1*01-DQA1*01:01-DQB1*05:01 (serotype DR1), DRB1*15-DQA1*01:02-DQB1*06:02-8 (serotype DR2), DRB1*11-DQA1*05:01-DQB1*03:01 (serotype DR5), DRB1*03-DQA1*05:01-DQB1*02 (serotype DR17), and DRB1*07-DQA1*02:01-DQB1*02 (serotype DR7). However, it was not possible to compare the haplotypes of the cohort of patients with those of a population control group. According to the literature, the DR1 serotype is associated with a high risk of HIV infection [45], DR17 determines the predisposition to type 1 diabetes mellitus, Sjögren’s syndrome, Graves’ disease [46], celiac disease [47], and DR5 determines the predisposition to juvenile idiopathic arthritis [48]. The DR7 serotype is predisposing to celiac disease [47] and protective against Graves’ disease [46], while the DR2 serotype protects against type 1 diabetes mellitus [49]. In the patients cohort we examined, there were no cases of the above diseases.

The association between HLA and COVID-19 deserves investigations on larger patient cohorts. The results of this study are based on observational data and analysis of patients in the “red zone” of the National Endocrinology Research Center, Moscow, Russia. This sample, of course, cannot characterize the population of patients with coronavirus infection as a whole. Additionally, the limitation of the present study is genotyping at the level of allele groups, not specific alleles. Moreover, only limited sets of HLA alleles as genetic markers of COVID-19 predisposition have been studied. More research is needed to access genetic changes that may play a deleterious role in the clinical course of COVID-19 patients, including analysis of the *ACE2* and *APOE* genes, major histocompatibility complex class I genes, Toll-like receptors, and others.

## 5. Conclusions

There was no association of sex, age, weight, body mass index, smoking, or comorbidity with COVID-19 severity in the patient cohort studied. Allele DQB1*06:02-8 was more common in patients (*p* < 0.00005), and DQB1*06:01 and DQB1*05:03 were more common in the control group (*p* < 0.00005, and *p* = 0.0011, respectively). DQB1*06:02-8 can probably be considered as predisposing to moderate and severe COVID-19, and DQB1*06:01 can be considered as protective. No association of these alleles with comorbidity was found. Further research on patients with mild and asymptomatic COVID-19 is required.

## Figures and Tables

**Table 1:** Diagnostic and anamnestic data of patients included in the study (data on several patients were not available due to an inaccuracy in the collection of anamnesis).

Clinical Feature	All Patients	Moderate COVID-19 (CT-1 or CT-2)	Severe COVID-19 (CT-3 or CT-4)	*p* (*p*_0_ *=* 0.003 after Bonferroni Correction)
Number of Patients	*n* = 100	*n* = 41	*n* = 59	
Duration of hospital stay, days	13 (6, 26) (median value (min, max) are presented for quantitative variables)	11 (6, 19)	14 (8, 26)	0.00002 (U)
Fatal outcome	3 (3%)	0	3 (5.1%)	0.267 (FET)
Age, years	58.5 (34, 96)	54 (36, 89)	61 (34, 96)	0.062 (U)
Sex, males	52 (52%)	18 (43.9%)	34 (57.6%)	0.223 (FET)
Age, years				
Females (*n* = 48)	67 (40, 96)	58 (40, 89)	71 (43, 96)	0.021 (U)
Males (*n* = 52)	51 (34, 88)	48 (36, 79)	57.5 (34, 88)	0.233 (U)
BMI, kg/m^2^	28.4 (16.9, 64)	26.4 (16.9, 42,2)	30.2 (20.8, 64)	0.017 (U)
Weight, kg	84.5 (45, 185)	77 (45, 180)	87 (58, 185)	0.007 (U)
Smoking	68	33	35	-
Smoking during hospitalization	5 (7%)	3 (9.1%)	2 (5.7%)	0.668 (FET)
Of them males	4 (80%)	2 (67%)	2 (100%)	1.000 (FET)
Invasive mechanical ventilation, number of patients, %	3 (3%)	0	3 (5.1%)	0.267 (FET)
Autoimmune diseases	16 (16%)	7 (17.1%)	9 (15.3%)	1.000 (FET)
Endocrine	8 (8%)	4 (9.8%)	4 (6.8%)	0.713 (FET)
Autoimmune thyroiditis	8 (8%)	4 (9.8%)	4 (6.8%)	0.713 (FET)
Nonendocrine	8 (8%)	3 (7.3%)	6 (10.2%)	0.734 (FET)
Bronchial asthma	5 (5%)	1 (2.4%)	4 (6.8%)	0.646 (FET)
Psoriasis/psoriatic arthritis	2 (2%)	1 (2.4%)	1 (1.7%)	1.000 (FET)
Rheumatoid arthritis	1 (1%)	0	1 (1.7%)	1.000 (FET)
Guillain–Barre disease	1 (1%)	0	1 (1.7%)	1.000 (FET)
Bechterew’s disease	1 (1%)	1 (2.4%)	0	0.410 (FET)
Non-autoimmune endocrine diseases	59 (59%)	21 (51.2%)	38 (64.4%)	0.218 (FET)
Obesity	45 (45%)	13 (31.7%)	32 (54.2%)	0.040 (FET)
Thyroid disease	15 (15%)	6 (14.6%)	9 (15.3%)	1.000 (FET)
Type 2 diabetes mellitus (according to the medical history (carbohydrate metabolism disorders first detected during hospitalization were not considered))	12 (12%)	5 (12.1%)	7 (11.9%)	1.000 (FET)
Diseases of the parathyroid glands	12 (12%)	5 (12.1%)	7 (11.9%)	1.000 (FET)
Others	1 (1%)	1 (2.4%)	0	0.410 (FET)
Cardiovascular diseases	64 (64%)	23 (56.1%)	41 (69.5%)	0.206 (FET)
Arterial hypertension	55 (55%)	20 (48.8%)	35 (59.3%)	0.315 (FET)
Coronary heart disease	21 (21%)	8 (19.5%)	13 (22.0%)	0.808 (FET)
Atherosclerosis of the aorta, lower limb arteries	12 (12%)	4 (9.8%)	8 (13.6%)	0.757 (FET)
Chronic heart failure	4 (4%)	2 (5%)	2 (3.4%)	1000 (FET)
History of acute violation of cerebral circulation	4 (4%)	3 (7.3%)	1 (1.7%)	0.302 (FET)
Heart rhythm disorders	3 (3%)	0	3 (5.1%)	0.267 (FET)
Others	1 (1%)	1 (2.4%)	0	0.410 (FET)
Oncological disorders	5 (5%)	1 (2.4%)	3 (5.1%)	0.642 (FET)
Chronic leukemia	2 (2%)	0	1 (1.7%)	1.000 (FET)
Breast cancer	2 (2%)	1 (2.4%)	1 (1.7%)	1.000 (FET)
Vulva cancer	1 (1%)	0	1 (1.7%)	1.000 (FET)
Lung diseases	26 (26%)	11 (17%)	16 (27.1%)	1.000 (FET)
Pulmonary hypertension	8 (8%)	1 (2.4%)	7 (11.9%)	0.136 (FET)
Emphysema	7 (7%)	4 (10%)	3 (5.1%)	0.441 (FET)
Previous tuberculosis	4 (4%)	4 (10%)	3 (5.1%)	0.441 (FET)
Chronic bronchitis	5 (5%)	3 (7.3%)	2 (3.4%)	0.398 (FET)
Bronchial asthma	5 (5%)	1 (2.4%)	4 (6.8%)	0.646 (FET)
Decreased lung volume due to injury/surgery	2 (2%)	0	2 (3.4%)	0.511 (FET)
Others	2 (2%)	1 (2.4%)	1 (1.7%)	1.000 (FET)

**Table 2:** HLA typing: frequencies of alleles of class II HLA genes in the entire cohort and in the CT groups.

Alleles	All Patients *n* = 100, 200 Alleles		Moderate COVID-19 (CT-1 or CT-2) *n* = 41, 82 Alleles	Severe COVID-19 (CT-3 or CT-4) *n* = 59, 118 Alleles	p *, χ^2^
	*n* (%)	95% CI for Proportion	*n* (%)	*n* (%)	
DRB1*01	28 (14%)	[9.5%; 19.6%]	15 (18.3%)	13 (11.0%)	0.145
DRB1*03 (DRB1*17)	20 (10%)	[6.2%; 15.0%]	10 (12.2%)	10 (8.5%)	0.388
DRB1*04	15 (7.5%)	[4.3%; 12.1%]	6 (7.3%)	9 (7.6%)	0.932
DRB1*07	24 (12%)	[7.8%; 17.3%]	9 (10.9%)	15 (12.7%)	0.689
DRB1*08	6 (3%)	[1.1%; 6.4%]	2 (2.4%)	4 (3.4%)	1.000 (FET)
DRB1*09	3 (1.5%)	[0.3%; 4.3%]	1 (1.2%)	2 (1.7%)	1.000 (FET)
DRB1*10	5 (2.5%)	[0.8%; 5.7%]	1 (1.2%)	4 (3.4%)	0.646 (FET)
DRB1*11	25 (12.5%)	[8.3%; 17.9%]	12 (14.6%)	13 (11%)	0.447
DRB1*12	8 (4%)	[1.7%; 7.7%]	2 (2.4%)	6 (5%)	0.466 (FET)
DRB1*13	21 (10.5%)	[6.6%; 15.6%]	9 (10.9%)	12 (10.2%)	0.846
DRB1*14	3 (1.5%)	[0.3%; 4.3%]	0	3 (2.5%)	0.270 (FET)
DRB1*15	33 (16.5%)	[11.6%; 22.4%]	10 (12.2%)	23 (19.5%)	0.172
DRB1*16	9 (4.5%)	[2.1%; 8.4%]	5 (6.1%)	4 (3.4%)	0.481 (FET)
DQA1*01:01	35 (17.5%)	[12.5%; 23.5%]	16 (19.5%)	19 (16.1%)	0.532
DQA1*01:02	39 (19.5%)	[14.3%; 25.7%]	12 (14.6%)	27 (22.9%)	0.148
DQA1*01:03	17 (8.5%)	[5.0%; 13.3%]	10 (12.2%)	7 (5.9%)	0.101
DQA1*02:01	24 (12%)	[7.8%; 17.3%]	9 (10.9%)	15 (12.7%)	0.710
DQA1*03:01	20 (10%)	[6.2%; 15.0%]	7 (8.5%)	13 (11.0%)	0.565
DQA1*04:01	4 (2%)	[0.6%; 5.0%]	2 (2.4%)	2 (1.7%)	1.000 (FET)
DQA1*05:01	60 (30%)	[23.7%; 36.9%]	26 (31.7%)	34 (28.8%)	0.660
DQA1*06:01	1 (0.5%)	[0.01%; 2.8%]	0	1 (0.8%)	1.000 (FET)
DQB1*02	38 (19%)	[13.8%; 25.1%]	15 (18.3%)	23 (19.5%)	0.832
DQB1*03:01	42 (21%)	[15.6%; 27.3%]	15 (18.3%)	27 (22.9%)	0.433
DQB1*03:02	13 (6.5%)	[3.5%; 10.9%]	6 (7.3%)	7 (5.9%)	0.685
DQB1*03:03	9 (4.5%)	[2.1%; 8.4%]	5 (6.1%)	4 (3.4%)	0.481 (FET)
DQB1*03:04	1 (0.5%)	[0.01%; 2.8%]	0	1 (0.8%)	1.000 (FET)
DQB1*03:05	1 (0.5%)	[0.01%; 2.8%]	1 (1.2%)	0	0.410 (FET)
DQB1*04:01/02	4 (2%)	[0.6%; 5.0%]	2 (2.4%)	2 (1.7%)	1.000 (FET)
DQB1*05:01	33 (16.5%)	[11.6%; 22.4%]	16 (19.5%)	17 (14.4%)	0.339
DQB1*05:02/04	10 (5%)	[2.4%; 9.0%]	5 (6.1%)	5 (4%)	0.736 (FET)
DQB1*05:03	1 (0.5%)	[0.01%; 2.8%]	0	1 (0.8%)	1.000 (FET)
DQB1*06:01	5 (2.5%)	[0.8%; 5.7%]	3 (3.7%)	2 (1.7%)	0.398 (FET)
DQB1*06:02-8	43 (21.5%)	[16.0%; 27.9%]	14 (17.1%)	29 (24.6%)	0.204

* *p*_0_ = 0.0015 after Bonferroni correction.

**Table 3:** Comparative analysis of frequencies of alleles of HLA class II genes in the examined cohort of patients and in the population control group.

	Frequency of Alleles in Patients Cohort	Number of Alleles in Patients Cohort	Frequency of Alleles in Control Group	Number of Alleles in Control Group	*p* *, Yates Corrected *χ^2^*
Number of Patients	*n* = 100		*n* = 327		-
Number of Alleles	*n* = 200		*n* = 654		-
DRB1*01	0.140	28	0.095	62	0.091
DRB1*03(DRB1*17)	0.100	20	0.075	49	0.322
DRB1*04	0.075	15	0.115	75	0.142
DRB1*07	0.120	24	0.143	94	0.463
DRB1*08	0.030	6	0.018	12	0.470
DRB1*09	0.015	3	0.007	5	0.599
DRB1*10	0.025	5	0.013	8	0.337
DRB1*11	0.125	25	0.142	93	0.617
DRB1*12	0.040	8	0.028	18	0.507
DRB1*13	0.105	21	0.142	93	0.217
DRB1*14	0.015	3	0.023	15	0.687
DRB1*15	0.165	33	0.132	86	0.280
DRB1*16	0.045	9	0.067	44	0.329
DQA1*01:01	0.175	35	0.130	85	0.137
DQA1*01:02	0.195	39	0.190	124	0.946
DQA1*01:03	0.085	17	0.107	70	0.442
DQA1*02:01	0.120	24	0.143	94	0.463
DQA1*03:01	0.100	20	0.122	80	0.463
DQA1*04:01	0.020	4	0.015	10	0.888
DQA1*05:01	0.300	60	0.290	189	0.833
DQA1*06:01	0.005	1	0.003	2	0.782
DQB1*02:01	0.190	38	0.195	128	0.939
DQB1*03:01	0.210	42	0.240	157	0.433
DQB1*03:02	0.065	13	0.083	54	0.510
DQB1*03:03	0.045	9	0.033	22	0.592
DQB1*03:04	0.005	1	0.002	1	0.958
DQB1*03:05	0.005	1	0.017	11	0.368
DQB1*04:01/02	0.020	4	0.022	14	0.873
DQB1*05:01	0.165	33	0.108	71	0.044
DQB1*05:02/04	0.050	10	0.032	21	0.333
DQB1*05:03	0.005	1	0.068	44	*0.0011*
DQB1*06:01	0.025	5	0.200	131	*<0.00005*
DQB1*06:02-8	0.215	43	0.000	0	*<0.00005*

* p_0_ = 0.0015 for DQB1 after Bonferroni correction.

**Table 4:** Characteristics of patients carrying presumably protective (DQB1*06:01) and presumably predisposing (DQB1*06:02-8, DQB1*05:03) alleles.

	DQB1*05:03	DQB1*06:01	DQB1*06:02-8
Number of Alleles	1	5	43
Number of Patients	1	5	35
Haplotypes	DRB1*14-DQA1*01:01-DQB1*05:03–1 (100%)	DRB1*15-DQA1*01:03-DQB1*06:01–5 (100%)	DRB1*15-DQA1*01:02-DQB1*06:02-8–27 (63%) DRB1*13-DQA1*01:03-DQB1*06:02-8–12 (28%) DRB1*13-DQA1*01:02-DQB1*06:02-8–3 (7%) DRB1*15-DQA1*03:01-DQB1*06:02-8–1 (2%)
Duration of hospital stay, days	12	13 (11, 19)	13 (7, 21)
Fatal outcome	0	0	1 (2.9%)
Age, years	34	47 (41, 65)	58 (34, 96)
Sex, male	1 (100%)	2 (40%)	21 (60%)
Age			
Females	-	43 (41, 47)	69 (42, 96)
Males	34	48; 65	51 (34, 83)
BMI, kg/m^2^	27.7	26.5 (22, 33,1)	27.4 (16.9, 41.6)
Weight, kg	82	80 (65, 99)	86.5 (45, 120)
Smoking Smoking during hospitalization Of them males	30 2 1	5 0 0	25 2 (8%) 1 (50%)
CT-1	0	0	2 (5.7%)
CT-2	0	3 (60%)	10 (28.6%)
CT-3	0	2 (40%)	17 (48.6%)
CT-4	1 (100%)	0	6 (17.1%)
Invasive mechanical ventilation	0	0	1 (2.9%)
Autoimmune diseases	0	1 (20%)	5 (14%)
Endocrine diseases	0	1 (20%)	3 (9%)
Autoimmune thyroiditis	0	1 (20%)	3 (9%)
Nonendocrine	0	0	3 (9%)
Bronchial asthma	0	0	3 (9%)
Psoriasis/ psoriatic arthritis	0	0	0
Rheumatoid arthritis	0	0	1 (2.9%)
Guillain–Barre disease	0	0	0
Bechterew’s disease	0	0	0
Non-autoimmune endocrine diseases	0	2 (40%)	18 (51%)
Obesity	0	1 (20%)	13 (37%)
Thyroid disease	0	1 (20%)	6 (17%)
Type 2 diabetes mellitus	0	0	4 (11%)
Diseases of the parathyroid glands	0	0	2 (5.7%)
Others	0	0	0
Cardiovascular diseases	0	0	21 (60%)
Arterial hypertension	0	0	17 (48.6%)
Coronary heart disease	0	0	6 (17.1%)
Atherosclerosis of the aorta, lower limb arteries	0	0	5 (14%)
Chronic heart failure	0	0	1 (2.9%)
History of acute violation of cerebral circulation	0	0	0
Heart rhythm disorders	0	0	1 (2.9%)
Others	0	0	0
Oncological diseases	0	0	4 (11%)
Chronic leukemia	0	0	2 (5.7%)
Breast cancer	0	0	1 (2.9%)
Vulva cancer	0	0	1 (2.9%)
Lung diseases	0	1 (20%)	9 (26%)
Pulmonary hypertension	0	1 (20%)	1 (2.9%)
Emphysema	0	0	2 (5.7%)
Previous tuberculosis	0	0	2 (5.7%)
Chronic bronchitis	0	0	2 (5.7%)
Bronchial asthma	0	0	3 (9%)
Decreased lung volume due to injury/surgery	0	0	0
History of pneumonia	0	0	1 (2.9%)

**Table 5:** HLA typing: frequencies of haplotypes of class II HLA genes in the entire cohort of patients and in the groups with moderate and severe COVID-19.

Haplotype	All Patients *n* = 100, 200 Alleles		Moderate COVID-19 (CT-1 or CT-2) *n* = 41, 82 Alleles	Severe COVID-19 (CT-3 or CT-4) *n* = 59, 118 Alleles	p *, Yates χ^2^
	*n* (%)	95% CI for Proportion	*n* (%)	*n* (%)	
DRB1*01-DQA1*01:01-DQB1*05:01	28 (14%)	[9.5%; 19.6%]	15 (18.3%)	13 (11.0%)	0.211
DRB1*03(DRB1*17)-DQA1*05:01-DQB1*02	20 (10%)	[6.2%; 15.0%]	10 (12.2%)	10 (8.5%)	0.533
DRB1*04-DQA1*03:01-DQB1*03:01	2 (1%)	[0.1%; 3.6%]	0	2 (1.7%)	0.644
DRB1*04-DQA1*03:01-DQB1*03:02	12 (6%)	[3.1%; 10.3%]	6 (7.3%)	6 (5.1%)	0.726
DRB1*04-DQA1*03:01-DQB1*03:04	1 (0.5%)	[0.01%; 2.8%]	0	1 (0.8%)	0.854
DRB1*07-DQA1*02:01-DQB1*02	18 (9%)	[5.42%; 13.82%]	6 (7.3%)	12	0.658
DRB1*07-DQA1*02:01-DQB1*03:03	6 (3%)	[1.1%; 6.4%]	4 (4.9%)	2 (1.7%)	0.381
DRB1*08-DQA1*03:01-DQB1*03:02	1 (0.5%)	[0.01%; 2.8%]	0	1 (0.8%)	0.854
DRB1*08-DQA1*04:01-DQB1*04:01/02	4 (2%)	[0.6%; 5.0%]	2 (2.4%)	2 (1.7%)	0.886
DRB1*08-DQA1*06:01-DQB1*03:01	1 (0.5%)	[0.01%; 2.8%]	0	1 (0.8%)	0.854
DRB1*09-DQA1*03:01-DQB1*03:03	3 (1.5%)	[0.3%; 4.3%]	1 (1.2%)	2 (1.7%)	0.750
DRB1*10-DQA1*01:01-DQB1*05:01	5 (2.5%)	[0.8%; 5.7%]	1 (1.2%)	4 (3.4%)	0.613
DRB1*11-DQA1*05:01-DQB1*03:01	25 (12.5%)	[8.3%; 17.9%]	12 (14.6%)	13 (11.0%)	0.587
DRB1*11-DQA1*05:01-DQB1*03:05	1 (0.5%)	[0.01%; 2.8%]	1 (1.2%)	0	0.854
DRB1*12-DQA1*05:01-DQB1*03:01	8 (4%)	[1.7%; 7.7%]	2 (2.4%)	6 (5.1%)	0.567
DRB1*13-DQA1*01:02-DQB1*06:02-8	3 (1.5%)	[0.3%; 4.3%]	0	3 (2.5%)	0.388
DRB1*13-DQA1*01:03-DQB1*06:02-8	12 (6%)	[3.1%; 10.3%]	7 (8.5%)	5 (4.2%)	0.339
DRB1*13-DQA1*05:01-DQB1*03:01	6 (3%)	[1.1%; 6.4%]	2 (2.4%)	4 (3.4%)	0.973
DRB1*14-DQA1*01:01-DQB1*05:03	1 (0.5%)	[0.01%; 2.8%]	0	1 (0.8%)	0.854
DRB1*14-DQA1*01:01-DQB1*05:02/04	1 (0.5%)	[0.01%; 2.8%]	0	1 (0.8%)	0.854
DRB1*14-DQA1*05:01-DQB1*03:01	1 (0.5%)	[0.01%; 2.8%]	0	1 (0.8%)	0.854
DRB1*15-DQA1*01:02-DQB1*06:02-8	27 (13.5%)	[9.1%; 19.0%]	7 (8.5%)	20 (16.9%)	0.133
DRB1*15-DQA1*01:03-DQB1*06:01	5 (2.5%)	[0.8%; 5.7%]	3 (3.7%)	2 (1.7%)	0.679
DRB1*16-DQA1*01:02-DQB1*05:02/04	9 (4.5%)	[2.1%; 8.4%]	5 (6.1%)	4 (3.4%)	0.574

* *p*_0_ = 0.002 after Bonferroni correction.

## Data Availability

The data presented in this study are available on request from the corresponding author.

## Supplementary Materials

The following supporting information can be downloaded at: https://www.mdpi.com/article/10.3390/diseases10040099/s1, Table S1: Clinical characteristics, anamnestic data and results of HLA typing for COVID-19 patients included in the study.
