# Supplementary material for: Association of Alleles of Human Leukocyte Antigen Class II Genes and Severity of COVID-19 in Patients of the ‘Red Zone’ of the Endocrinology Research Center, Moscow, Russia
Source: Diseases. 2022 Nov 2;10(4):99. doi: 10.3390/diseases10040099 (PMC9680278; doi:10.3390/diseases10040099)
Supplement: Supplementary file 1 [file diseases-10-00099-s001.zip › diseases-1959793-supplementary.pdf]

Table S1. Clinical characteristics, anamnestic data and results of HLA typing for COVID-19 patients included in the study.

№	Age, years	Sex	BMI, kg / m2	Weight, kg	Smoking (yes / no)	Outcome (improvement/ fatal)	IMV (yes/no)	Duration of hospital stay, days	tomography during hospitalization	Maximum lung CT lesion	Group (1 -, moderate, 2 – severe)	Endocrine AID	Nonendocrine AID	ED of nonautoimmune origin	CVD	OD	LD	CRP at admission, mg/L	SARS-CoV-2 viral RNA at	SpO2 at admission, %	DRB1 - allele 1	DRB1 - allele 2	DQA1 - allele 1	DQA1 - allele 2	DQB1 - allele 1	DQB1 - allele 2
1	80	f	35,2	90	no	improvement	no	11	2	4	2	AT	no	obesity, nodular goiter	AH	no	history of tuberculosis, emphysema	197,6	neg	99	03	16	05:01	01:02	02	05:02/05:04
2	89	f	23,4	60	no	improvement	no	12	2	2	1	no	no	no	AH, CHF, CHD, ACVA	no	pulmonary hypertension	74,2	neg	89	07	13	02:01	05:01	02	03:01
3	64	m	25,4	75	no	improvement	no	16	3	3	2	no	psoriasis	no	CHD, aortic atherosclerosis	no	no	128,8	pos	96	04	07	03:01	02:01	03:01	02
4	48	m	22	65	no	improvement	no	12	2	2	1	no	no	no	no	no	no	81,3	neg	95	13	15	01:03	01:03	06:02-8	06:01
5	46	m	26,2	94	no	improvement	no	15	3	3	2	no	no	no	no	no	no	62,8	pos	94	11	15	05:01	01:02	03:01	06:02-8
6	70	f	35,2	90	no	improvement	no	14	1	2	1	no	no	obesity, secondary hyperparathyroidism	AH, ACVA	no	no	19,8	neg	99	11	16	05:01	01:02	03:01	05:02/05:04
7	76	m	24,7	73	no	improvement	no	14	3	3	2	no	no	no	CHD, RBBB	no	emphysema	58,3	pos	95	15	15	01:02	01:02	06:02-8	06:02-8
8	66	f	25,4	65	no	improvement	no	14	3	2	1	no	no	secondary hyperparathyroidism	AH, CHD	no	no	36,2	pos	99	07	12	02:01	05:01	03:03	03:01
9	80	m	26,	72	N	improvem	n	26	6	3	2	no	no	no	AH, CHF,	no	history of	80,5	ne	96	11	16	05:0	01:0	03:01	05:02/05:0

			8		D	ent	o								CHD		tuberculosis		g				1	2		4
10	71	m	25,4	70	no	improvement	no	20	4	3	2	no	Guillain-Barre disease	no	AH	no	chronic bronchitis, post-traumatic reduction of the left lung sizes with areas of pulmonary fibrosis, pulmonary hypertension	144,4	neg	92	07	11	02:01	05:01	02	03:01
11	83	m	23,5	64	no	improvement	no	10	1	3	2	no	no	no	AH	no	no	141,9	neg	94	11	13	05:01	01:02	03:01	06:02-8
12	55	f	30,8	77	no	improvement	no	11	2	2	1	no	no	DM2, obesity, nodular goiter	AH, CHD	no	no	63,4	pos	93	04	07	03:01	02:01	03:02	03:03
13	50	f	42,2	108	ND	improvement	no	10	2	2	1	no	no	obesity	no	no	no	95,6	pos	92	04	07	03:01	02:01	03:02	02
14	51	m	23	70	no	improvement	no	11	2	2	1	no	no	no	no	no	chronic bronchitis, history of tuberculosis	7,1	neg	40	03	13	05:01	01:03	02	06:02-8
15	40	m	30	95	no	improvement	no	8	2	1	1	no	no	obesity, DM2	AH	no	no	21,8	neg	91	04	11	03:01	05:01	03:02	03:01
16	51	m	26,6	102	no		no	14	2	3	2	no	no	no	AH	no	resection of the lower lobe of the left lung for a history of spontaneous pneumothorax, pulmonary hypertension, emphysema	259,5	pos	95	03	11	05:01	05:01	02	03:01

17	61	f	31,6	83	no	improvement	no	14	2	3	2	no	BA	obesity, diffuse goiter	AH	no	BA	14,4	neg	88	11	16	05:01	01:02	03:01	05:02/05:04
18	54	f	64	185	no	improvement	no	13	2	3	2	no	no	obesity, DM2	AH	no	no	64,7	pos	88	01	01	01:01	01:01	05:01	05:01
19	71	f	26,2	74	no	improvement	no	13	2	2	1	AT	no	nodular goiter	AH	no	no	58,7	pos	99	01	03	01:01	05:01	05:01	02
20	65	m	26,5	84	no	improvement	no	19	3	3	2	no	no	no	no	no	no	80,1	pos	92	04	15	03:01	01:03	03:02	06:01
21	75	f	37,5	90	no	fatal	yes	20	1	4	2	no	no	DM2, obesity	CHD, aortic atherosclerosis	no	no	73,1	pos	92	07	15	02:01	01:02	02	06:02-8
22	54	f	23,2	61	no	improvement	no	7	2	2	1	no	no	no	no	no	no	61,9	neg	98	04	11	03:01	05:01	03:02	03:05
23	62	f	28	70	no	improvement	no	13	2	2	1	no	Bechterew's disease	DM2	CHD, AH	BC	emphysema	13,7	pos	93	01	11	01:01	05:01	05:01	03:01
24	59	f	31,6	85	ND	improvement	no	15	2	3	2	no	no	obesity	no	no	pulmonary hypertension	79,5	pos	96	03	07	05:01	02:01	02	02
25	73	f	32	82	ND	improvement	no	11	1	3	2	no	no	obesity, secondary hyperparathyroidism	AH	no	no	57,1	neg	84	01	01	01:01	01:01	05:01	05:01
26	49	m	33	105	no	improvement	no	13	3	4	2	no	no	obesity	no	chronic leukemia	no	58,2	pos	90	04	13	03:01	01:03	03:01	06:02-8
27	42	f	23,7	67	no	improvement	no	9	2	2	1	no	BA	no	no	no	BA	6,1	neg	95	11	15	05:01	01:02	03:01	06:02-8
28	40	f	29,8	85,1	ND	improvement	no	12	2	2	1	no	no	no	no	no	no	8,9	neg	92	01	03	01:01	05:01	05:01	02
29	75	f	39	90	ND	improvement	no	12	2	3	2	no	no	obesity	AH, ACVA	no	chronic bronchitis	1,5	neg	97	01	04	01:01	03:01	05:01	03:02
30	48	m	33,1	105	no	improvement	no	6	2	2	1	no	no	obesity	AH	no	no	13,9	pos	95	01	11	01:01	05:01	05:01	03:01
31	46	f	30,3	94	no	improvement	no	15	2	3	2	no	no	obesity	no	no	no	287,3	pos	93	03	04	05:01	03:01	02	03:02

32	66	f	33,7	79	no	improvement	no	11	1	1	1	no	no	Cushing's disease, obesity , DM2	AH, CHD, CHF	no	subpleural lipoma	2,1	neg	97	07	16	02:01	01:02	02	05:02/05:04
33	41	f	28	80	no	improvement	no	13	2	2	1	AT	no	multinodular goiter	no	no	no	29,6	pos	97	03	15	05:01	01:03	02	06:01
34	85	f	30,2	68	no	improvement	no	15	1	3	2	AT	no	obesity	AH	no	pulmonary hypertension	114,4	neg	95	04	13	03:01	05:01	03:02	03:01
35	37	m	24,5	76	ND	improvement	no	14	4	3	2	no	no	no	AH	no	no	194,9	pos	95	01	11	01:01	05:01	05:01	03:01
36	38	m	24,1	73	no	improvement	no	7	1	1	1	no	no	no	no	no	no	5,5	neg	99	11	13	05:01	01:03	03:01	06:02-8
37	43	f	30,1	82	no	improvement	no	20	3	3	2	no	no	obesity	no	no	no	188,6	neg	88	01	15	01:01	01:02	05:01	06:02-8
38	82	m	34	90	ND	improvement	no	18	3	4	2	no	no	obesity	CHD, Frederick's syndrome	no	no	92,5	pos	76	11	16	05:01	01:02	03:01	05:02/05:04
39	53	f	20,4	55	no	improvement	no	10	2	2	1	no	psoriatic arthritis	no	lower limb atherosclerosis	no	no	166,2	pos	94	01	10	01:01	01:01	05:01	05:01
40	63	m	28,1	87	no	improvement	no	13	2	2	1	no	no	no	AH	no	no	13,9	pos	92	12	15	05:01	01:02	03:01	06:02-8
41	43	f	33,1	99	no	improvement	no	13	2	3	2	no	no	obesity	no	no	pulmonary hypertension	56,5	pos	95	07	15	02:01	01:03	02	06:01
42	60	m	28,7	89	no	improvement	no	11	2	1	1	no	no	nodular goiter	AH, aortic atherosclerosis	no	no	13,4	neg	98	01	11	01:01	05:01	05:01	03:01
43	70	f	41,6	116	ND	improvement	no	18	4	3	2	no	no	obesity	AH	no	no	255,4	neg	92	13	13	01:03	01:03	06:02-8	06:02-8
44	71	f	20,8	58	no	improvement	no	15	3	3	2	no	no	secondary hyperparathyroidism, nodular goiter	AH, CHD	no	no	44,7	pos	99	01	10	01:01	01:01	05:01	05:01
45	46	m	36,3	112,5	ND	improvement	no	14	1	2	1	no	no	obesity	AH	no	no	23,9	pos	92	13	15	01:03	01:02	06:02-8	06:02-8
46	51	f	27,3	79	no	improvement	no	11	3	2	1	no	no	no	AH	no	no	1,7	neg	95	13	15	01:03	01:02	06:02-8	06:02-8

47	57	f	23,7	67	no	improvement	no	10	1	2	1	no	no	nodular goiter	no	no	no	51,3	pos	90	08	15	04:01	01:02	04:01/04:02	06:02-8
48	53	m	23,8	79	ND	improvement	no	11	2	3	2	no	no	no	no	no	no	34,6	pos	92	03	08	05:01	03:01	02	03:02
49	47	m	32,9	102	no	improvement	no	12	2	3	2	no	no	obesity	no	no	no	14,6	pos	96	07	08	02:01	04:01	02	04:01/04:02
50	42	m	24,6	78	ND	improvement	no	12	2	2	1	no	no	no	no	no	no	13,8	pos	99	01	03	01:01	05:01	05:01	02
51	47	f	23	68	no	improvement	no	11	2	2	1	no	no	no	no	no	no	31,8	pos	95	04	15	03:01	01:03	03:02	06:01
52	35	m	23	85	ND	improvement	no	13	2	3	2	no	no	no	no	no	no	133,4	neg	92	03	03	05:01	05:01	02	02
53	71	m	30,5	85	yes	improvement	no	12	2	2	1	no	no	obesity	AH, CHD	no	emphysema	80,2	neg	93	01	11	01:01	05:01	05:01	03:01
54	59	m	37	120	ND	improvement	no	18	2	4	2	no	no	DM2, obesity	AH	no	no	53,1	neg	82	13	15	01:02	01:02	06:02-8	06:02-8
55	83	f	32	92	ND	fatal	yes	20	2	4	2	no	no	obesity, secondary hyperparathyroidism	AH	no	no	143,4	pos	93	07	11	02:01	05:01	02	03:01
56	58	m	38,7	120	no	improvement	no	13	2	3	2	no	no	obesity	AΓ, CHD, aortic atherosclerosis	no	no	84,9	pos	97	12	15	05:01	01:02	03:01	06:02-8
57	60	m	34,3	111	ND	improvement	no	11	2	3	2	no	no	DM2, obesity	AH	no	no	237,8	pos	95	13	13	01:03	05:01	06:02-8	03:01
58	52	m	26,2	85	no	improvement	no	10	2	2	1	no	no	secondary hyperparathyroidism	AH	no	no	85,9	pos	99	03	11	05:01	05:01	02	03:01
59	75	f	30,8	87	no	improvement	no	18	3	4	2	no	no	DM2, obesity	AH	no	no	152,7	pos	92	10	11	01:01	05:01	05:01	03:01
60	55	m	30,2	100	no	improvement	no	15	2	4	2	no	no	obesity	no	no	no	38,9	pos	95	01	15	01:01	01:02	05:01	06:02-8
61	51	m	36,2	112	no	improvement	no	13	1	2	1	no	no	obesity	no	no	chronic bronchitis	94,1	neg	99	08	13	04:01	01:03	04:01/04:02	06:02-8

62	62	f	29,7	79	no	improvement	no	15	3	4	2	no	no	nodular goiter	no	no	pulmonary hypertension	139,3	pos	91	13	15	01:03	01:02	06:02-8	06:02-8
63	46	m	28,7	95	ND	improvement	no	10	2	2	1	no	no	no	no	no	no	139,6	neg	93	01	15	01:01	01:02	05:01	06:02-8
64	45	m	35,5	180	no	improvement	no	10	1	2	1	no	no	obesity, secondary hyperparathyroidism	pulmonary artery embolism.	no	no	22,1	pos	91	16	16	01:02	01:02	05:02/05:04	05:02/05:04
65	96	f	38	96	no	improvement	no	10	2	3	2	AT	BA	obesity, nodular goiter	AH, CHD , CHF	no	BA, history of tuberculosis	25,9	pos	95	15	15	01:02	01:02	06:02-8	06:02-8
66	59	m	26,8	85	no	improvement	no	11	3	3	2	no	no	no	CHD	no	no	50,9	pos	94	07	07	02:01	02:01	02	02
67	80	f	36,6	102	ND	improvement	no	15	1	3	2	no	no	obesity, nodular goiter, secondary hyperparathyroidism	AH, CHD, aortic atherosclerosis	no	no	79,4	pos	93	11	12	05:01	05:01	03:01	03:01
68	59	f	20,9	57	no	improvement	no	13	3	2	1	no	no	no	no	no	no	77,1	pos	97	01	07	01:01	02:01	05:01	02
69	38	m	31,4	95	yes	improvement	no	10	1	3	2	no	no	obesity	AH	no	no	25,8	neg	98	04	07	03:01	02:01	03:02	02
70	57	f	25	64	no	improvement	no	14	2	2	1	no	no	no	no	no	no	34,2	pos	94	07	09	02:01	03:01	02	03:03
71	58	m	26,4	80	ND	improvement	no	23	3	4	2	no	BA	no	AH	no	BA	66,7	neg	95	03	04	05:01	03:01	02	03:02
72	35	m	23,7	90	yes	improvement	no	14	4	3	2	no	no	no	AH	no	no	232,1	pos	92	12	15	05:01	01:02	03:01	06:02-8
73	58	f	26,4	71	no	improvement	no	9	1	2	1	no	no	no	no	no	no	82,2	neg	97	03	03	05:01	05:01	02	02
74	45	m	30,9	100	no	improvement	no	10	3	3	2	no	no	obesity	no	no	no	89,1	pos	97	08	13	04:01	05:01	04:01/04:02	03:01
75	34	m	27,7	82	ND	improvement	no	12	2	4	2	no	no	no	no	no	no	250,6	neg	85	03	14	05:01	01:01	02	05:03
76	81	f	27	64	ND	improvement	no	17	4	3	2	no	RA, BA	secondary hyperparathyroidis	AH	no	BA	125,1	pos	92	04	15	03:01	03:01	03:04	06:02-8

														m												
77	68	f	26	75	ND	improvement	no	12	1	3	2	no	no	DM2, multinodular goiter	AH	no	no	76,8	pos	92	03	15	05:01	01:02	02	06:02-8
78	48	m	39,9	118	ND	improvement	no	13	2	3	2	no	no	obesity	AH	no	no	22,5	pos	97	08	11	06:01	05:01	03:01	03:01
79	36	m	33,9	98	no	improvement	no	14	1	1	1	no	no	obesity	no	no	no	203,5	neg	94	01	11	01:01	05:01	05:01	03:01
80	78	f	24	70	ND	improvement	no	13	2	2	1	AT	no	no	AH, CHD, aortic atherosclerosis	no	emphysema	79,5	pos	92	07	15	02:01	01:02	03:03	06:02-8
81	34	m	29,6	96	no	improvement	no	12	2	3	2	no	no	no	no	no	no	226,8	pos	90	13	15	01:02	01:02	06:02-8	06:02-8
82	67	m	32	94	ND	fatal	yes	24	2	3	2	no	no	obesity	AH	no	no	270,4	pos	91	10	12	01:01	05:01	05:01	03:01
83	79	m	23	69	ND	improvement	no	11	2	1	1	no	no	no	AH, CHD, ACVA, lower limb atherosclerosis	no	emphysema	67,5	pos	87	03	07	05:01	02:01	02	03:03
84	36	m	24	71	yes	improvement	no	9	2	2	1	no	no	no	no	no	no	65,1	pos	94	01	01	01:01	01:01	05:01	05:01
85	50	f	30,6	86,5	no	improvement	no	8	2	3	2	no	no	primary hyperparathyroidism, obesity, nodular goiter	AH	BC	no	37,9	neg	96	13	15	05:01	01:02	03:01	06:02-8
86	73	f	30,1	82	no	improvement	no	8	2	2	1	no	no	obesity, nodular goiter	AH	no	no	12,4	pos	95	11	13	05:01	05:01	03:01	03:01
87	70	f	25,3	69	ND	improvement	no	14	2	3	2	AT	no	no	AH, CHD, aortic atherosclerosis	VC	no	180,3	pos	93	11	15	05:01	01:02	03:01	06:02-8
88	60	m	24,5	75	no	improvement	no	8	1	2	1	no	no	secondary hyperparathyroidis	AH	no	no	1,9	pos	99	01	04	01:01	03:01	05:01	03:02

														m												
89	72	f	31	95	ND	improvement	no	14	3	3	2	no	no	DM2, obesity	AH	no	no	151,5	pos	91	01	07	01:01	02:01	05:01	02
90	78	m	23,9	65	ND	improvement	no	21	3	4	2	no	no	no	lower limb atherosclerosis	chronic leukemia	no	294,4	pos	87	12	15	05:01	01:02	03:01	06:02-8
91	58	m	33,9	90	no	improvement	no	15	4	3	2	no	no	obesity	AH	no	no	10,7	pos	92	01	15	01:01	01:02	05:01	06:02-8
92	71	f	38	90	ND	improvement	no	18	3	2	1	AT	no	DM2, obesity	AH	no	chronic bronchitis	40,4	pos	93	01	03	01:01	05:01	05:01	02
93	67	m	23	65	no	improvement	no	11	3	3	2	no	no	nodular goiter	AH	no	no	45,2	neg	95	14	15	01:01	01:02	05:02/05:04	06:02-8
94	71	f	16,9	45	yes	improvement	no	19	2	1	1	no	no	no	MI	no	no	31,1	neg	84	13	16	01:03	01:02	06:02-8	05:02/05:04
95	68	f	28	82	ND	improvement	no	10	1	3	2	no	no	no	AH, CHD, aortic atherosclerosis	no	pulmonary hypertension	32,2	neg	96	01	07	01:01	02:01	05:01	03:03
96	88	m	26,3	70	no	improvement	no	12	2	3	2	no	no	secondary hyperparathyroidism	AH, CHD, AF, aortic atherosclerosis	no	no	90,2	neg	94	09	14	03:01	05:01	03:03	03:01
97	41	m	30,9	98	ND	improvement	no	11	1	3	2	no	no	obesity	no	no	no	138,1	pos	95	01	09	01:01	03:01	05:01	03:03
98	57	m	24,5	65	no	improvement	no	10	1	3	2	no	no	no	no	no	no	44,8	neg	96	07	10	02:01	01:01	02	05:01
99	43	m	27,4	97	no	improvement	no	13	3	3	2	no	no	no	no	no	history of the right-sided pneumonia	229,5	pos	91	07	15	02:01	01:02	03:03	06:02-8
100	72	f	32	87	no	improvement	no	16	3	3	2	no	no	obesity	AH	no	no	121,6	pos	90	07	12	02:01	05:01	02	03:01

Designations: f - female; м -male; ND – no data; IMV – invasive mechanical ventilation; CT – computer tomography; AID – autoimmune diseases; AT – autoimmune thyroiditis; ED – endocrine diseases; BA –bronchial asthma; RA – rheumatoid arthritis; DM2 –diabetes mellitus type 2; CVD – cardiovascular diseases; AH – arterial hypertension; CHF – chronic heart failure; AF – atrial fibrillation; CHD – coronary heart disease; RBBB – right bundle branch block; ACVA – acute cerebrovascular accident;

OD – oncological diseases; BC – breast cancer; LD – lung diseases; CRP – C-reactive protein; SARS-CoV-2 severe acute respiratory syndrome coronavirus 2; RNA – ribonucleic acid; MI – myocardial infarction; VC- vulvar cancer; neg – negative; pos - positive.
